# Wave-like Robotic Locomotion between Highly Flexible Surfaces and Comparison to Worm Robot Locomotion

**DOI:** 10.3390/biomimetics8050416

**Published:** 2023-09-07

**Authors:** Dan Shachaf, Rotem Katz, David Zarrouk

**Affiliations:** Department of Mechanical Engineering, Ben Gurion University of the Negev, Beersheba 8410501, Israelzadavid@bgu.ac.il (D.Z.)

**Keywords:** crawling robot, wave-like mechanism, undulating mechanism, minimally actuated, bioinspired robotics, flexible surface

## Abstract

In a recent study, we developed a minimally actuated robot that utilizes wave-like locomotion and analyzed its kinematics. In this paper, we present an analysis of the robot’s locomotion between two highly flexible surfaces. Initially, we created a simulation model of the robot between two surfaces and determined its speed and the conditions of locomotion based on the flexibility of the surface, the geometrical parameters, and the coefficient of friction for horizontal locomotion and climbing at different angles. Our findings indicate that wave locomotion is capable of consistently advancing along the surface, even when the surface is highly flexible. Next, we developed an experimental setup and conducted multiple experiments to validate the accuracy of our simulation. The results indicate an average relative difference of approximately 11% between the speed and advance ratio of the wave crawling between the two surfaces of our simulation model and the experimental results were performed using an actual robot. Lastly, we compared the wave locomotion results to those of the worm locomotion and discovered that wave locomotion outperforms worm locomotion, especially at a higher surface flexibility.

## 1. Introduction

Numerous studies have focused on bioinspired crawling robots in recent decades, as they can be utilized for a wide range of applications, including maintaining pipelines and crawling inside the body’s biological vessels. These robots must be capable of crawling over anisotropic and flexible terrains with varying surface properties and must respond flexibly to different coefficients of friction and dimensions, as noted in various studies [1,2,3,4]. A minimalistic approach is commonly utilized to achieve efficient design, which involves a small number of motors and actuators. Three types of locomotion have been explored in these studies: screw-like locomotion [5,6], worm-like locomotion [7,8,9,10,11,12,13,14,15,16,17,18,19,20,21,22,23,24,25,26,27,28] and undulating locomotion, which mimics a continuously advancing wave [29,30,31,32,33,34,35,36,37,38,39,40,41,42,43].

Among these locomotion types, robots employing undulating locomotion have demonstrated superior speeds compared to those based on screw-like or worm-like locomotion. Notably, certain worm-like robots, such as FabricWorm [27] and Meshworm [28], have achieved impressive maximum speeds of 0.55 mm/s and 0.506 mm/s, respectively. In contrast, a screw-propelling capsule robot has exhibited an average speed of 60 mm/s when maneuvering through a rubber pipe filled with water. However, these speeds are significantly slower when compared to the two fastest wave-like robots, the SAW and AmphiSAW. The SAW robot achieved a maximum speed of 57 cm/s when crawling over a rigid surface [29], and its amphibious version, the AmphiSAW, boasts a remarkable maximum ground speed of 77 cm/s when crawling on a polypropylene fabric solely relying on its wave-like mechanism [44].

Drawing inspiration from the wave-like locomotion observed in snakes and flagella, as well as the swimming patterns of miniature organisms, we previously introduced the Single Actuated Wave robot (SAW) [29]. SAW generates its wave motion by employing a rotating helix in combination with numerous links that are connected to one another via rotational joints. The robot’s simple design enables miniaturization down to a few centimeters in length, making it suitable for various medical applications [39,40]. Further studies have investigated the advantages of this unique propulsion mechanism in various other fields, including search and rescue, construction, and excavation. These studies have involved connecting multiple SAW robots [41,42,43] or utilizing the robot’s swimming abilities for applications as an amphibious robot [44] such as operating in proximity to wildlife for the monitoring, excavation and filming of natural reserves.

In the field of bioinspired robotics, there is extensive utilization of flexible materials. This utilization is seen in the design of actuators based on their flexibility [45]. It is also applied to the manufacturing of robots whose motion relies on compliant legs [46]. Furthermore, entirely flexible robots have been developed [47]. The widespread use of compliant materials presents challenges in accurately predicting and simulating the behaviors of robots due to the viscoelastic nature and nonlinearity of these flexible materials. To effectively model and simulate the behaviors of such flexible materials, advanced tools such as deep-learning-based point cloud techniques can be employed [48].

**Figure 1:**
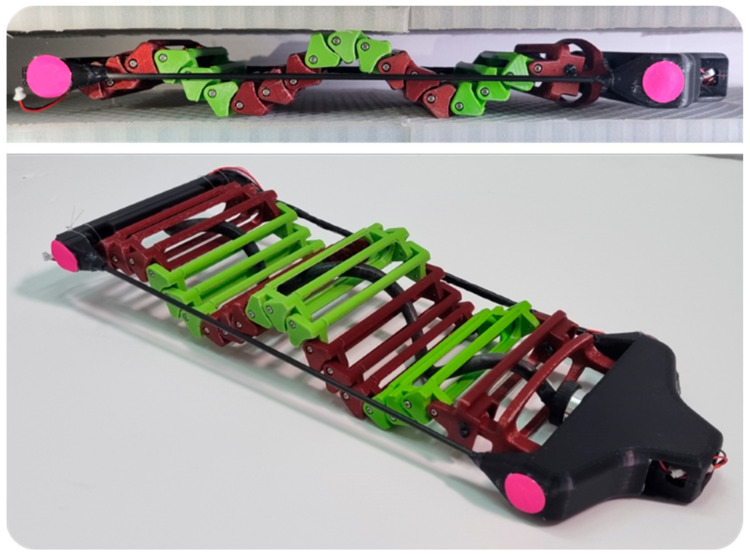
The SAW robot advances by generating an advancing wave that is actuated using a single motor. (**Top**) A side view of the robot crawling between highly flexible surfaces. (**Bottom**) An isometric view of the SAW robot.

The objective of this paper is to investigate the locomotion of the SAW robot in a highly flexible environment (Figure 1). Specifically, we aim to examine the robot’s ability to generate thrust and advance over both horizontal and inclined surfaces where climbing is required. There are two types of rigidities: structural flexibility, in which the surface is bendable, and contact flexibility, where the surface is flexible only at the contact point, regardless of whether the surface is bendable or not. Our focus, in this work, is on investigating the impact of contact flexibility on locomotion. In Section 2, we provide a brief description of the new robot design. Section 3 presents an analysis of the forces in action when the robot crawls between two surfaces. In Section 4, we present a new simulation of the robot crawling between two flexible surfaces, and the simulation results are reported in Section 5. Subsequently, in Section 6 we introduce the validation experiments, our simulation, and its results. In Section 7, we compare the robot’s locomotion to worm locomotion. Finally, we present our conclusions in Section 8.

## 2. Robot Design

In this section, we briefly describe the design of the experimental wave-producing robot as well as the manufacturing materials and electronic parts. For more details, please see [29].

### 2.1. The Robot’s Structure

**Figure 2:**
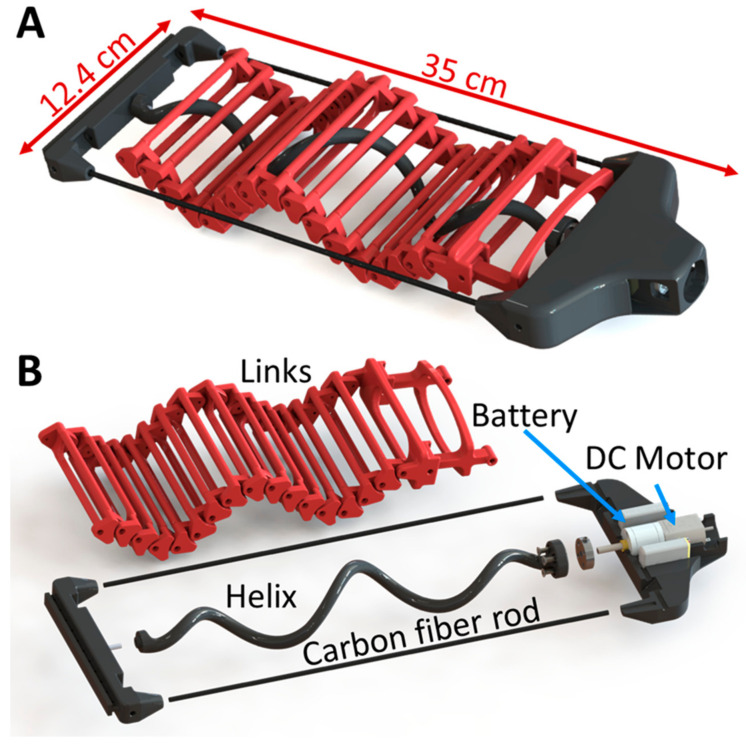
(**A**) The SAW robot. (**B**) An exploded view of the robot showing its main components: a rigid frame body, a DC motor, a rotating helix, and multiple links connected by rotating joints forming the waveform.

The SAW robot has a very simple design comprising three primary components: a head, a rigid body, and a tail that produces a wave-like motion (Figure 2). The head houses both the motor and batteries, and the body serves as the rigid framework supporting the head and tail. The tail consists of numerous links (20 in total—including two special links connecting the wave to the head) interconnected by rotating joints, with a helix that can rotate within the links to generate the wave motion. The robot’s length spans 35 cm, and its width measures 12.4 cm. The helix has a wavelength of 10.2 cm and an amplitude of 3.7 cm.

The robot is propelled by a single 6–8 volt DC motor, measuring 20 mm in diameter, which rotates the helix through a 156:1 gearbox. As the helix rotates, the links cancel out the rotational motion, and instead maintain vertical oscillation, effectively representing a sagittal projection of the helix. The robot’s direction of motion is solely dictated by the motor’s rotation direction, thereby enabling easy reversibility.

To minimize the effects of swinging and unwanted turns during its locomotion, a rigid frame was incorporated into the robot’s design, resulting in a sturdier structure.

### 2.2. Materials and Properties

The SAW robot has an efficient design that is easily reproducible on various scales. Weighing in at a total of 313 g, including the motor and batteries, or 296 g without the batteries, the SAW robot is a lightweight yet highly reliable robot with hardly any maintenance required. Comprised almost entirely of 3D-printed parts, with the exception of its motors, electronics, screws, and rigid carbon fiber bars, the robot’s production is simple and cost effective. We used a Tiertime UP300 MEM (Melted Extrusion Modelling) printer and PLA printing material. The density of various robot components depends on the loads they are subjected to, aiming to reduce the overall weight of the robot. The helix is 3D-printed with 100% density, as lower density tends to result in its fragility. The links are 3D-printed at 50% density, as this density allows them to be sufficiently robust to withstand forces and prevent breakage, while simultaneously reducing their weight. Other robot components experience lower stress during movement, allowing the use of a printing density of 30%. After printing, the helix was polished using sandpaper to decrease its friction with the links. After printing and cleaning the parts, the total assembly requires nearly one hour. The robot has rigid carbon fiber rods on each side to increase its rigidity against torsional bending.

## 3. Force Analysis of Crawling between Two Surfaces

**Figure 3:**
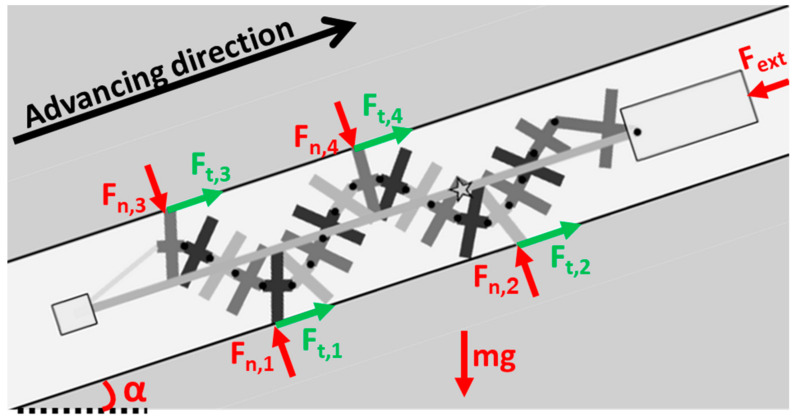
Free body diagram of the robot between two rigid surfaces as it climbs a slope in the presence of an external resisting force. The star represents the COM of the robot.

When a robot advances between two surfaces along a slope with an angle of α, it typically contacts the surface at four contact points, as shown in Figure 3. In order to move forward, the contact points must generate sufficient thrust to overcome the weight components *mg* and other resisting forces *F_ext_* (such as tether forces, for example). At each contact point “*i*”, a normal force *F*_*n*,*i*_ and a tangential force *F*_*t*,*i*_ act on it. In this specific example, the bottom contact points are labeled “1” and “2”, and the top contact points are labeled “3” and “4”. At low speeds and accelerations, the sum of the forces in the normal and tangential directions (including weight components and other resisting forces) must be zero.

The sum of the forces in the normal direction is:

(1) $$F_{n,1}+F_{n,2}=mg\cdot \cos\alpha +F_{n,3}+F_{n,4}$$

and the condition for the robot progress is:

(2) $$F_{t,1}+F_{t,2}+F_{t,3}+F_{t,4}>F_{ext}+mg\cdot \sin\alpha$$

Assuming there are identical coefficients of friction (COF—denoted by *μ*) for all the contact points, Equation (2) becomes:

(3) $$F_{n,1}+F_{n,2}+F_{n,3}+F_{n,4}>\frac{F_{ext}+mg\cdot \sin\alpha }{\mu }$$

Inserting (1) into (3) yields a condition for locomotion as a function of the normal bottom forces only:

(4) $$F_{n,1}+F_{n,2}>\frac{1}{2}\left[\frac{F_{ext}}{\mu }+mg\left(\frac{\sin\alpha }{\mu }+\cos\alpha \right)\right]$$

Alternatively, the condition of the locomotion can be presented as a function of the normal forces with the upper surface:

(5) $$F_{n,3}+F_{n,4}>\frac{1}{2}\left[\frac{F_{ext}}{\mu }+mg\left(\frac{\sin\alpha }{\mu }-\cos\alpha \right)\right]$$

**Figure 4:**
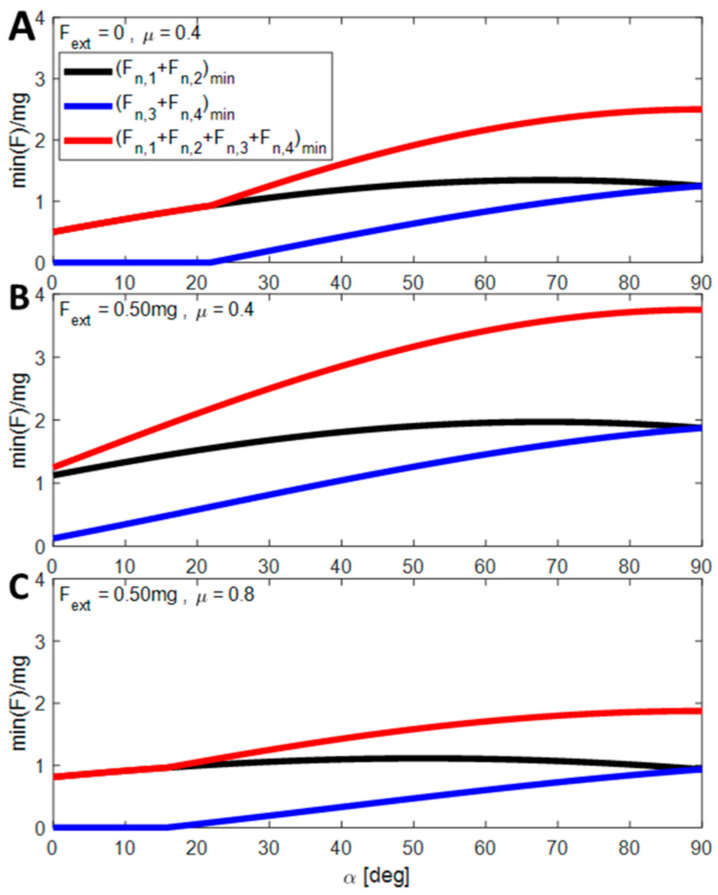
The sum of the minimal normal forces (bottom *F_n_*_,1_ + *F_n_*_,2_), (top, *F_n_*_,3_ + *F_n_*_,4_) and (total *F_n_*_,1_ + *F_n_*_,2_ + *F_n_*_,3_ + *F_n_*_,4_) required for the robot to advance as a function of the climbing slope for 3 different external forces *F_ext_* and COF conditions. (**A**) *F_ext_* = 0; μ = 0.4. (**B**) *F_ext_* = 0.5 mg; *μ* = 0.4. (**C**) *F_ext_* = 0.5; *μ* = 0.8.

Figure 4 presents the condition of locomotion (minimum values of the bottom normal forces) as a function of the slope for three different cases (A: *F_ext_* = 0; *μ* = 0.4), (B: *F_ext_* = 0.5 mg; *μ* = 0.4) and (C: *F_ext_* = 0.5; *μ* = 0.8). In (A), the robot can climb slopes up to 22 degrees without the support of the top layer. The required force increases gradually and reaches its maximum (2.5 mg) at 90 degrees (when the robot is climbing vertically). In (B), the robot needs the support of the top layer due to the resisting force acting on it. In this case, the maximum forces are also obtained at 90 degrees, when the normal and bottom forces are equal. In (C), the robot can climb slopes without the assistance of the top layer up to 15 degrees, despite facing a similar resisting force as in scenario (B). However, beyond 15 degrees, the top layer becomes necessary due to the higher coefficient of friction.

Figure 4 determines whether the top layer is necessary for climbing and provides the minimum required forces for the task. However, this problem is mechanically indeterminate, meaning that in order to solve it, we must know the compliance (elastic properties) of the surface to evaluate each of the normal forces. Therefore, we developed a multibody numerical simulation, which is presented in Section 4.

## 4. Simulation of Robot Crawling between Two Flexible Surfaces

**Figure 5:**
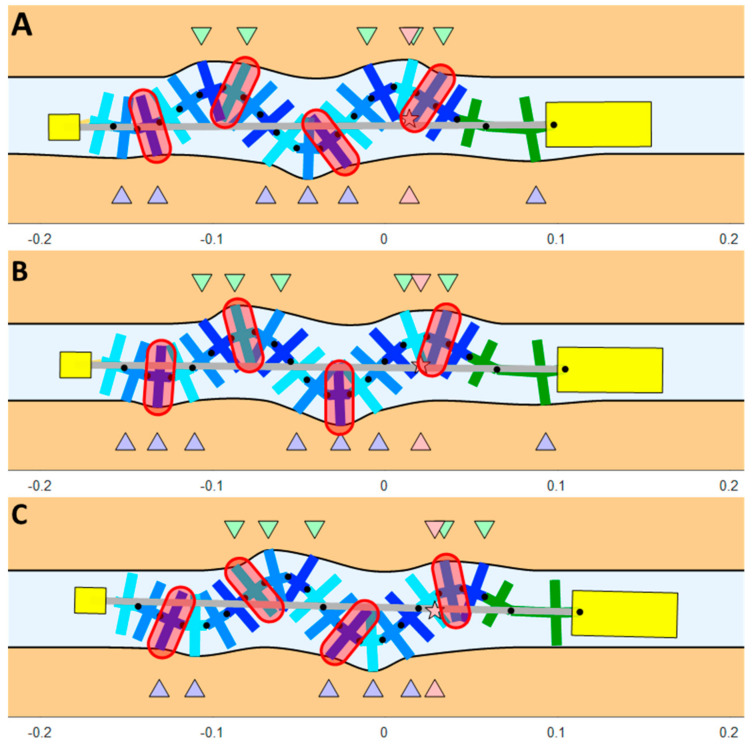
Simulation of the robot’s movement between two flexible surfaces (see Video S1). (**A**) Beginning of a step, (**B**) middle of the step, (**C**) end of the step. The purple and green arrows indicate the contact points between the robot and the bottom and upper surfaces, respectively. The pink star represents the robot’s COM, and the pink arrows represent its projection on the surfaces.

We created a 2D multibody simulation of the robot using MATLAB (see Figure 5). This simulation dynamically simulates the robot’s motion while taking into account factors such as the normal and tangential rigidity of the surface, the coefficient of friction, the distance between the two surfaces, the climbing slope, the external resisting force, the weight and inertia moment of all the robot parts, and the speed of the wave. By running the simulation, we can anticipate the robot’s behavior under various conditions and optimize its parameters for better performance.

### 4.1. Kinematic Modeling of the Robot

**Figure 6:**
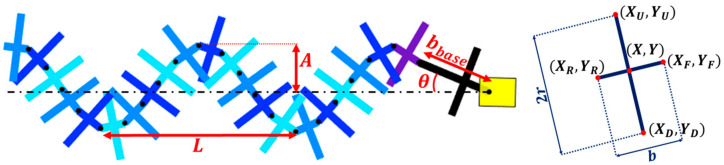
Modeling the robot. (**Left**) The geometrical parameters of the wave. (**Right**) Defining each of the links by five points, one at the center and four at its tips.

We begin our model by finding the position and orientation of the different links which perform a sinusoidal motion. The base (first) link is attached on one side to the motor head and on the second side, to the wave. The angle of the base link *θ_base_* satisfies the following equation:

(6) $$\sin\theta_{base}=\frac{A}{b_{base}}\cos\left(\frac{2\pi }{L}b_{base}\cos\theta_{base}-\omega t\right)$$

where *A* is the amplitude of the wave, *b_base_* is the width of the (first) base link, attached to the head of the robot, *L* is the wavelength, *ω* is the angular velocity of the helix and *t* is time (0). Wavelength, *ω,* is the angular velocity of the helix and *t* is time (Figure 6).

We define each link by five points—one at the middle of the link and the other four at the tips of the link (up, down, front, and rear). The front and rear points are the joins connecting consecutive links. The position and orientation of the links relative to the main body are geometrically calculated using (A1)–(A6) (see Appendix A).

The simulation can detect collisions between the links (allowing improved design) as well as the penetration of the links in the surrounding flexible surfaces.

### 4.2. Surface Modeling

At each contact point of the robot with the surface, there are two types of forces—normal and tangential. The Hertz model (which we use here for simplicity) assumes that these forces do not influence each other. The normal force, *F_n_*, is a function of the deformation, *δ_n_*, raised to the power of 1.5. The tangential force, *F_t_*, is proportional to the tangential deformation, *δ_t_*.

(7) $$F_{n}=k_{n}\cdot \delta_{n}{}^{1.5}$$

and

(8) $$F_{t}=k_{t}\cdot \delta_{t}$$

Because the simulation continuously calculates the coordinates of the robot, the surfaces, and the links, we can calculate the displacement of the surfaces and their derivatives over time. We can also apply a coefficient of restitution (COR) to the normal force in case there is any energy dissipation during locomotion. Please note that a small hysteresis was observed during the experiments. By accounting for this hysteresis, we can calculate the normal forces as follows:

(9) $$F_{n}=\left\{\begin{array}{cc} k_{n}\cdot \delta_{n}{}^{1.5}, & {\dot{\delta }}_{n}>0\\ e\cdot k_{n}\cdot \delta_{n}{}^{1.5}, & {\dot{\delta }}_{n}\leq 0 \end{array}\right.$$

where ${\dot{\delta }}_{n}$ is the rate of deformation of the surface.

When considering the tangential direction, we must take into account the surface coefficient of friction (COF) and sliding conditions. Therefore, it is calculated as follows:

(10) $$F_{t}=\min\left\{\begin{array}{l} k_{t}\delta_{t}\\ \mu F_{n} \end{array}\right.$$

Equation (8) indicates that the tangential force increases linearly as the link drags the surface during its paddling-like motion until it reaches the same magnitude as the maximum friction force *μF_n_*. At this point, any further movement causes the link to slide, as the tangential force remains constant.

### 4.3. Multibody Simulation

As we described in Section 3, we define our coordinates in the tangential and normal direction to the surface in its initial form (positive to the right). The simulation calculates the robot’s motion by solving three equations: the sum of forces acting on the robot’s COM in the normal and tangential directions yields its accelerations in these directions ($\ddot{x}$, $\ddot{y}$), whereas the sum of moments (*M*) around the third axle at the robot’s COM yields the rotational acceleration of the robot $\ddot{\theta }$.

(11) $$\left\{\begin{array}{l} \sum_{i}F_{t,i}=m\cdot \ddot{x}\\ \sum_{i}F_{n,i}=m\cdot \ddot{y}\\ \sum_{i}M_{i}=\sum_{i}r_{i}\times F_{i}=I\cdot \ddot{\theta } \end{array}\right.$$

where *m* and *I* are the robot’s mass and moment of inertia, respectively, **r** is the distance vector between the COM and the force acting point, and **F** is the force vector.

The simulation evaluates the robot’s motion by dividing it into discrete time steps (*dt*). Based on the forces at each contact point, it calculates the linear and angular acceleration of the robot in every time step. This allows the simulation to calculate the linear and angular velocities ($\dot{x},\dot{y},\dot{\theta }$) of the robot and its position and orientation (*x, y, θ*), by integrating it numerically, as follows:

(12) $$\left\{\begin{array}{l} {\dot{x}}_{i}={\ddot{x}}_{i}\cdot dt+{\dot{x}}_{i-1}\\ x_{i}={\dot{x}}_{i}\cdot dt+x_{i-1} \end{array}\right.$$

We ran our simulation at 1000 Hz after we found that it numerically converges to an accurate solution. The algorithm of the simulation is presented as a pseudo code in Appendix A.2.

## 5. Simulation Results

**Figure 7:**
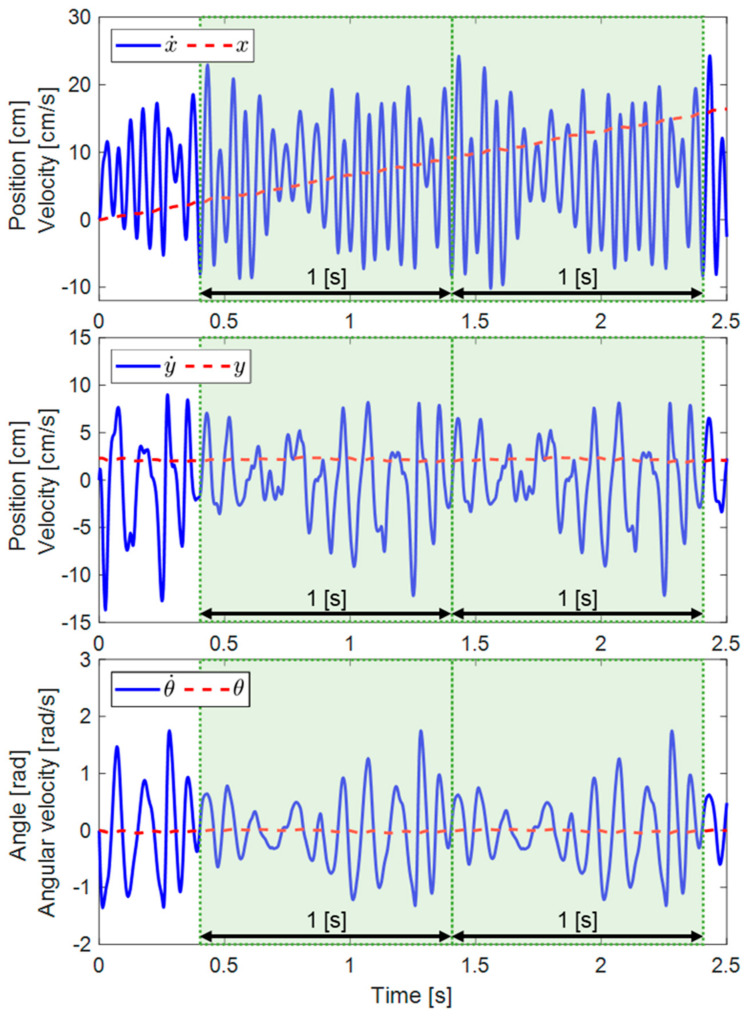
The position and velocity of the robot in the tangential and normal directions, along with its pitch and angular velocity, were simulated with a motor frequency set to 1 Hz.

The basic outputs of the simulation are the position and velocities of the robot along the tangential and normal axes, as well as its pitch and angular velocity. Figure 7 presents the simulation’s results during the first 2.5 s of the robot’s motion starting from rest and actuated at 1 Hz (i.e., one wave cycle per second).

**Figure 8:**
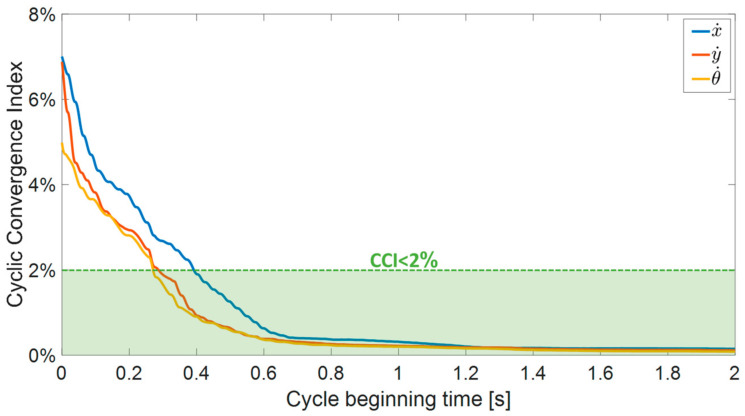
The calculated cyclic convergence index (CCI) used to assess the convergence of the simulation outcome based on the robot’s velocities.

When the beginning of the movement is in transition mode, the robot’s velocities quickly settle to a steady state. In order to evaluate the convergence of simulation outcomes towards a steady state, an analysis was conducted to determine the variances between the initial and subsequent motion cycles. We defined a cyclic convergence index (CCI) as a dimensionless parameter used to quantify the absolute disparities between two consecutive cycles and assess the convergence of the simulation outcome. The CCI is calculated as follows:

(13) $$CCI=\frac{\sum \left|v_{i}-v_{i+1/dt}\right|}{\sum \left|v_{i}-v_{avg}\right|}$$

where *v_i_* and *v_i_*_+1/*dt*_ represent the instantaneous velocity of the robot in two consecutive cycles, and *v_avg_* is its average velocity, calculated from the steady-state phase of the motion. The CCI for the first two seconds of the simulation outcome is presented in Figure 8, indicating that all the velocities are converging (CCI > 2%) after 0.4 s and reaching below 0.2% after 1.2 s.

To compare the results of experiments performed under different conditions, we defined a dimensionless quantity AR (advance ratio), which is the ratio of the robot’s velocity in the tangential direction (the proceeding direction) divided by the theoretical velocity of the robot when crawling over a rigid surface:

(14) $$AR=\frac{v_{robot}}{v_{theory}}$$

The theoretical velocity of the robot over a rigid surface was calculated by [29] using:

(15) $$v_{theory}=4\pi^{2}\frac{A}{L}rf$$

### 5.1. Advancing Ratio as a Function of the Slope

**Figure 9:**
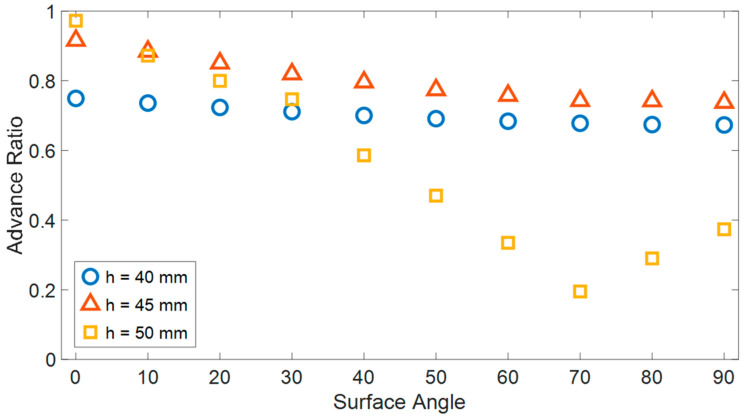
The advance ratio of the robot (AR) for three different distances between the two surfaces (40, 45 and 50 mm) as a function of the surface’s angle.

To examine the effect of surface inclination on robot AR, a simulation was conducted to replicate the robot’s movement across angles spanning from 0 to 90 degrees (Figure 9). It is expected that the influence of inclination is contingent upon the normal forces exerted between the robot and the surfaces, i.e., the magnitude of the gap separating the two surfaces. Therefore, the simulation was executed for three distinct distances between the surfaces (40, 45, and 50 mm).

A greater distance between surfaces allows for higher AR in horizontal locomotion but hinders the robot’s motion on steeper slopes. For example, with a distance of 50 mm, the AR of the robot’s planar motion approaches unity but significantly decreases as the inclination angle increases.

On the other hand, reducing the distance between surfaces increases the normal forces, enabling the robot to achieve improved climbing performance, especially when facing larger surface angles. For a distance of 40 mm, the variation in surface inclination angle had a minimal impact on the robot’s AR. However, the AR was lower compared to larger surface distances, particularly for relatively small inclinations (up to approximately 30 degrees). Out of the three simulations, the 45 mm distance between surfaces represents an optimal intermediate solution that balances multiple advantages and results in relatively efficient robot motion that only marginally decreases with increasing inclinations.

### 5.2. Influence of the Flexibility for Different Surface Distances

**Figure 10:**
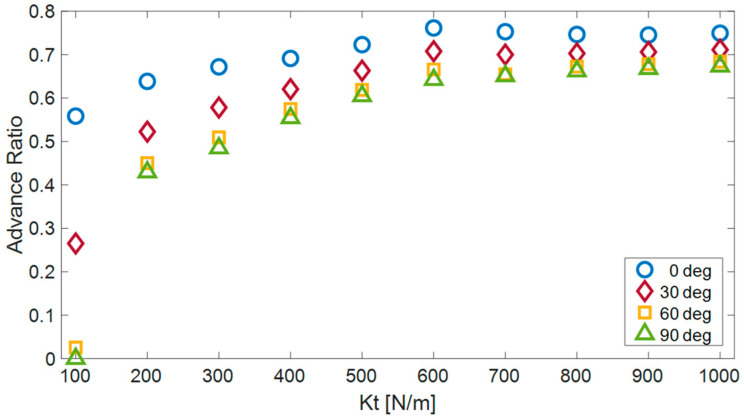
The advance ratio of the robot (AR) for four different orientations (0, 30, 60 and 90 degrees) as a function of the tangential compliance.

To investigate the effect of surface flexibility on the efficiency of robot motion, simulations of the robot’s movement were conducted on surfaces with varying flexibilities (ranging from 100 to 1000 N/m) and inclinations within the range of 0 to 90 degrees (see Figure 10). Consistent with our expectations, it was observed that the efficiency of robot motion improves with an increase in surface rigidity, enabling a more effective utilization of the robot’s link motion for propulsion while minimizing surface distortion.

Analysis of the simulation results revealed that, for flexibilities below 600 N/m (0.6 N/mm), an increase in surface rigidity substantially enhances the AR. Conversely, for higher flexibilities, the influence of surface rigidity on the efficiency of robot motion becomes less significant. Moreover, notable disparities were observed in the efficiency of the robot when climbing across different inclinations, particularly when dealing with surfaces of lower rigidity. These disparities diminish as the rigidity of the surfaces increases.

### 5.3. Influence of the COF on Crawling

The COF dictates the maximum tangential forces generated by the robot’s motion, resulting from the interaction between its links and the surface. One can expect that increasing the COF will enhance the efficiency of robot motion and increase its speed, although the relationship is somewhat more complex. Because the robot has multiple contact points with the ground, each moving at different velocities, contact forces opposing the robot’s forward motion are generated. Raising the COF amplifies these forces as well. Therefore, increasing the COF does not necessarily lead to an overall improvement in the robot’s AR.

**Figure 11:**
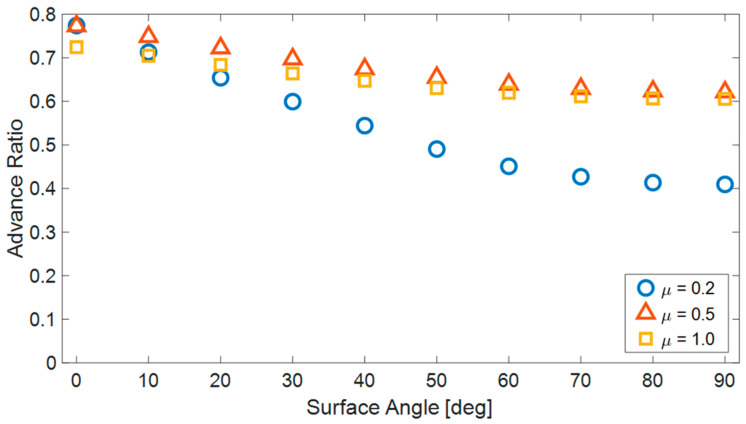
The advance ratio of the robot (AR) for three different COFs (0.2, 0.5 and 1.0) as a function of the surface’s angle.

The simulation results (Figure 11) demonstrate that, in the case of planar motion, a lower COF allows for slightly more efficient movement. However, when the surface inclination angles increase, a COF of 0.2 significantly decreases the efficiency of robot motion. On the other hand, for higher coefficients of friction (0.5 and 1.0), the inclination angle has little significant impact on the efficiency of robot motion. A coefficient of friction of 0.5 enables the most efficient motion across all ranges of surface inclinations.

## 6. Validation Experiments and Results

In order to verify and validate the simulation results, we have devised an experimental setup comprising several key components. Firstly, there is the robot itself, which will be interacting with the experimental environment. Secondly, the experimental environment consists of two planes which can be adjusted to vary the distance between them. On these planes, we have attached silicone rubber flexible surfaces which have been specifically designed with unique patterns to produce high compliance.

To accurately measure the surface properties of these flexible surfaces, we have also designed an indentation system. This system allows us to determine both the friction and stiffness of the surfaces, which are critical factors in understanding their behavior during interaction with the robot. Finally, a tracking system has been developed to measure the speed of the robot as it moves across the surfaces.

### 6.1. Manufacturing a Synthetic Flexible Surface

**Figure 12:**
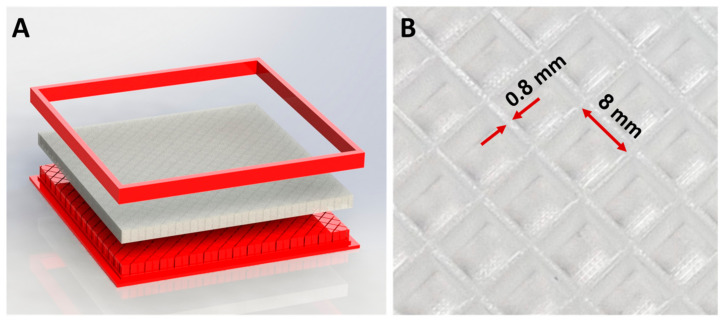
(**A**) The synthetic surface and its 3D-printed mold. (**B**) The surface’s hollow squares are rotated by 45° relative to the direction of locomotion.

The synthetic surfaces were created using a Dragon Skin^TM^ 10 NV silicone rubber cast which was converted into 3D-printed molds. These surfaces were hollow squares that were rotated by 45 degrees in relation to the direction of crawling, with an edge length of 8 mm and a thickness of 0.8 mm (see Figure 12). We selected this shape because it is relatively simple to produce and ensures consistent behavior, reducing the impact of the contact point’s location regarding the surface geometry. Each of the molds had a square shape measuring 20 × 20 cm, which was limited by the printer’s volume. The curing of the surface requires nearly 2 h, and the removal of the molds was performed manually by disassembling the mold’s outer frame and gently peeling off the silicone surface.

### 6.2. Contact Properties Measurement

**Figure 13:**
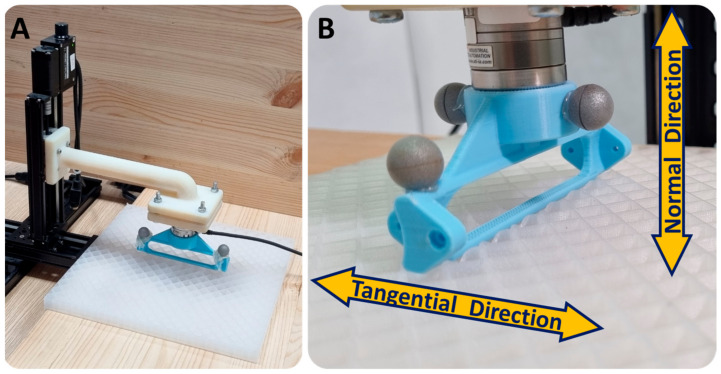
The surface stiffness measurement system is composed of a linear stage and a Nano 25 force sensor (**A**). Infrared makers are attached to the link to measure its position as the linear stage moves in the normal and tangential directions (**B**).

The forces that act between the robot and the surface are dependent on the stiffness of the surface in both the normal and tangential directions. To measure this stiffness, we designed an experimental system (see Figure 13) consisting of two linear stage actuators (with an accuracy of 60 µm when unloaded), a Nano 25 force sensor (with a 300 N sensing range and a 0.0625 N resolution), and a link-shaped indenter. However, as the linear stage actuators tend to bend under force, we used an OptiTrack tracking system (12 Prime 13 cameras with a 1.3 MP resolution) to monitor the position of the indenter during these experiments.

**Figure 14:**
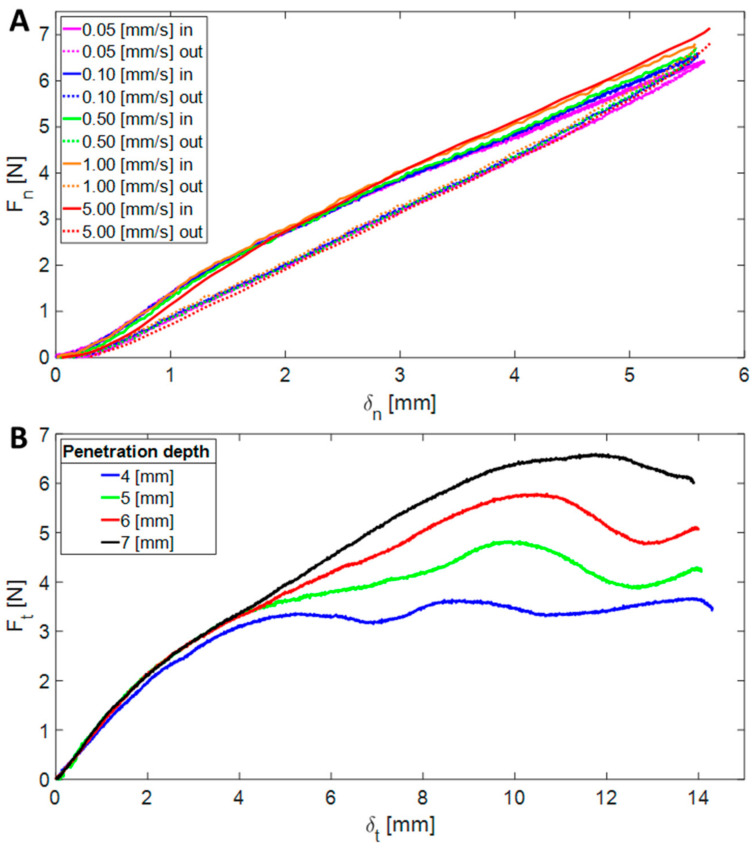
The results of the indentation experiment. The normal forces as a function of the penetration depth at various velocities (**A**), and the tangential forces vs. the tangential movement of the indenter in different penetration depths (**B**).

In the first experiment, the link-shaped indenter was moved downwards by 6 mm into the surface and back up. This experiment was repeated at various velocities ranging from 0.05 to 5 mm/s. From these experiments (see Figure 14A), we discovered that the stiffness is independent of the indentation rate and has a value of approximately k_n_ = 1200 N/m.

The second experiment was designed to measure the tangential stiffness of the surface and its coefficient of friction (COF). In each step of this experiment, the indenter moved down to a different penetration depth (4, 5, 6, and 7 mm), and then moved 15 mm forward parallel to the surface to ensure that slipping occurred (Figure 14B). The indenter’s velocity during this experiment was 1 mm/s. From the results of this experiment, we measured the tangential stiffness of the surface as k_t_ = 1082 N/m. These results also indicated that the slipping term depended on the penetration level of the indenter, as expected. The COF was calculated by dividing the tangential force by the normal force, and its value was found to be μ = 1.01.

Based on the results of these analyses, we performed multiple experiments where the robot was made to crawl between two highly flexible surfaces that were custom-made for these experiments.

### 6.3. Experimental System

**Figure 15:**
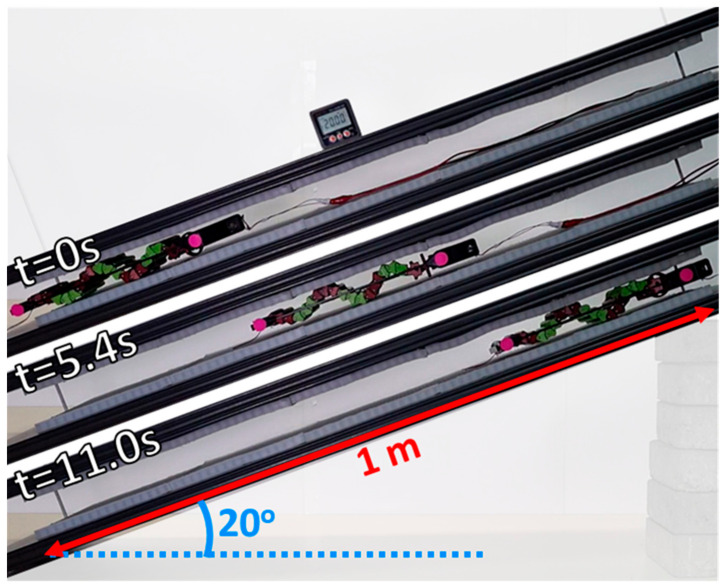
A side view of the experimental setup. In this specific experiment, the robot crawled over a 20-degree incline. Starting at *t* = 0, it finished climbing within 11 s, with an average speed of 6.8 cm/s.

The experimental system consisted of two parallel rigid boards that were 100 cm in length. We affixed five molded surfaces to each of the boards, covering the entire length of the boards. This allowed the robot to crawl through multiple cycles and reach a steady state of crawling. The spacing between the two surfaces was adjustable, enabling the application of different levels of pressure and allowing for the manipulation of normal forces acting on the robot (see Figure 15). Two pink markers were placed on the robot, one at its front and the other at its back. The experiments were recorded on video, and an offline image processing technique was employed (using MATLAB) to estimate the robot’s position (and velocity) during its motion. This was carried out at a rate of 60 fps, allowing for nearly 60 measurements per robot wave cycle.

### 6.4. Crawling Experiments and Comparison to Simulations

In this section, we present the experiments that we performed using our experimental setup and compare them to the simulation results to validate our simulation.

#### 6.4.1. Crawling Experiments

The robot was actuated between the flexible surfaces of the experimental setup, while systematically altering the slopes of the surface and the supplied electrical voltage (to vary its actuation frequency). The aim was to assess how these variations (slope and actuation frequency) influenced the robot’s crawling speed and its AR. The experiments were conducted with a fixed distance of 45 mm between the flexible surfaces, encompassing slopes ranging from −20 to 20 degrees (in steps of 10 degrees) and voltage levels of 4, 5, 6 and 7 V. Figure 15 presents an experiment carried out with a 20-degree angle and a 6 V operating voltage. In this trial, the robot exhibited an average speed of approximately 6.8 cm/s and completed the track within 11 s.

**Figure 16:**
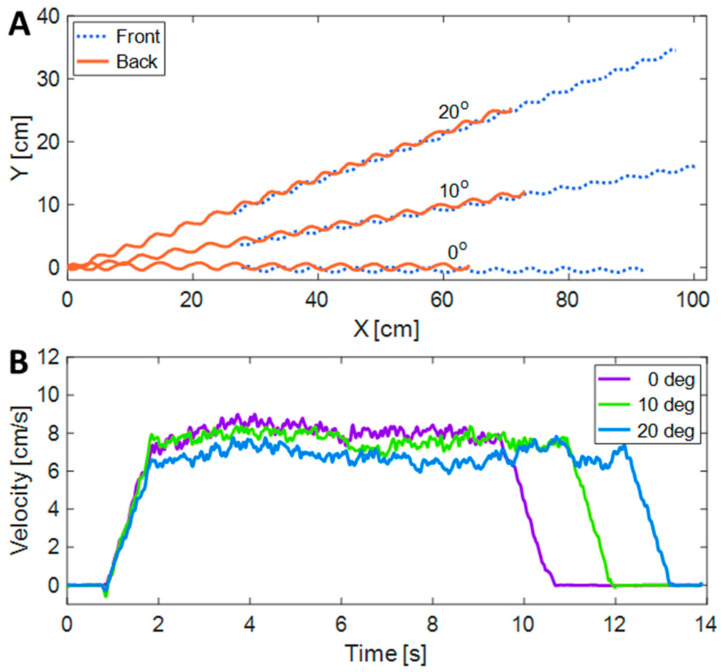
The positions of the front and back markers for three different angles, as recorded through video post-processing (**A**) and their velocities in the proceeding direction (**B**).

Two pink markers were applied to the robot, one on its front and the other on its rear section (see Figure 15). The markers’ positions were captured through video footage and subsequently analyzed using an image processing algorithm to estimate the robot’s position, velocity, and pitch angle. In Figure 16, the positions and velocities of the markers can be observed as the robot moves across three different inclinations (0, 10, and 20 degrees).

**Figure 17:**
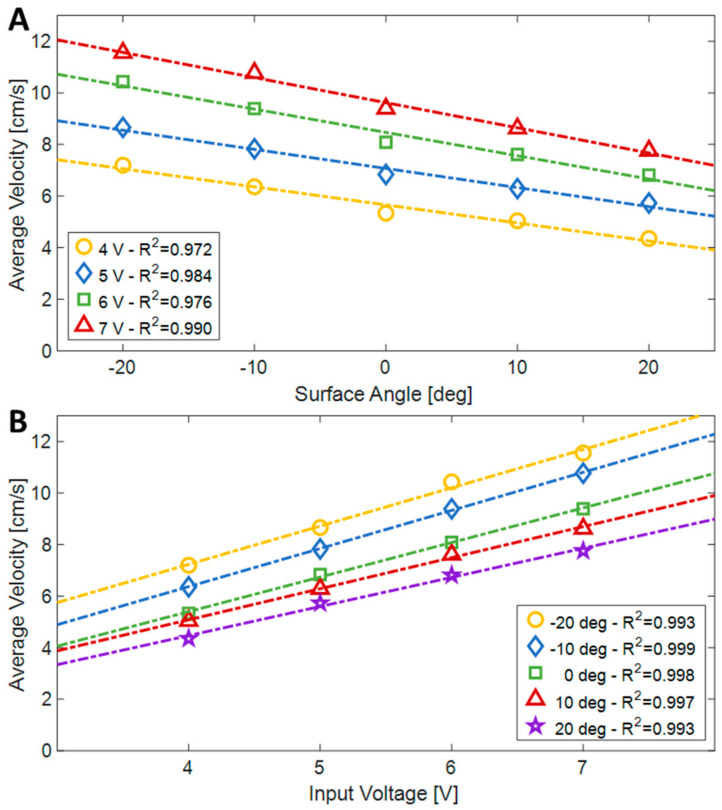
The average velocity of the robot as a function of the surface angle (**A**) and the input voltage (**B**).

The results of the experiments are summarized in Figure 17. The speeds ranged from 4.35 cm/s when climbing a 20-degree slope with a 4 V input, to 11.55 cm/s when descending the 20-degree slope with a 7 V input. Although there was no direct control, the speed of the robot was constant in each experiment and decreased almost linearly with the slope (R^2^ ≥ 0.972), whereas it linearly increased as a function of the applied voltage (R^2^ ≥ 0.993).

#### 6.4.2. Comparison to Simulations

**Figure 18:**
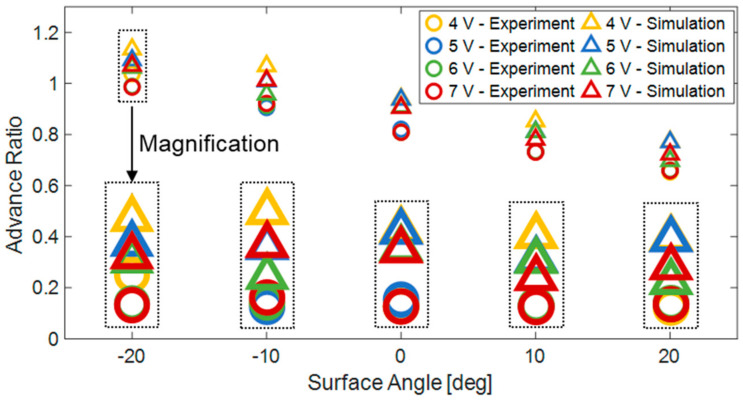
AR comparison between 20 simulation results (triangles) and experiments (circles) for different slope angles and input voltages.

To validate the simulation results, we performed 20 simulations and compared them to the experiments. Figure 18 compares the average values of the robot’s AR for the different experiments and simulations across various surface slopes and robot operating voltages. It can be observed that the approximation accuracy improves with higher operating voltages. At a 4 V operating voltage, there is an average relative error of 15.41%, compared to the average relative errors of 8.05% and 9.32% observed at 6 V and 7 V operating voltages, respectively. It can be inferred that the discrepancy between the experimental results and the simulation is due to certain simplifications made during the development of the simulation, such as assuming infinite rigidity of robot links and the actuator. Additionally, in the simulation, the motor rotates at a uniform speed without deceleration due to variable contact points and internal friction. Therefore, the simulation is more similar to the robot’s behavior at higher operating voltages, where the motor can cope with resistance without slowing down, unlike lower operating voltages where external loads may decelerate the motor’s rotation.

## 7. Comparison to Worm Locomotion

**Figure 19:**
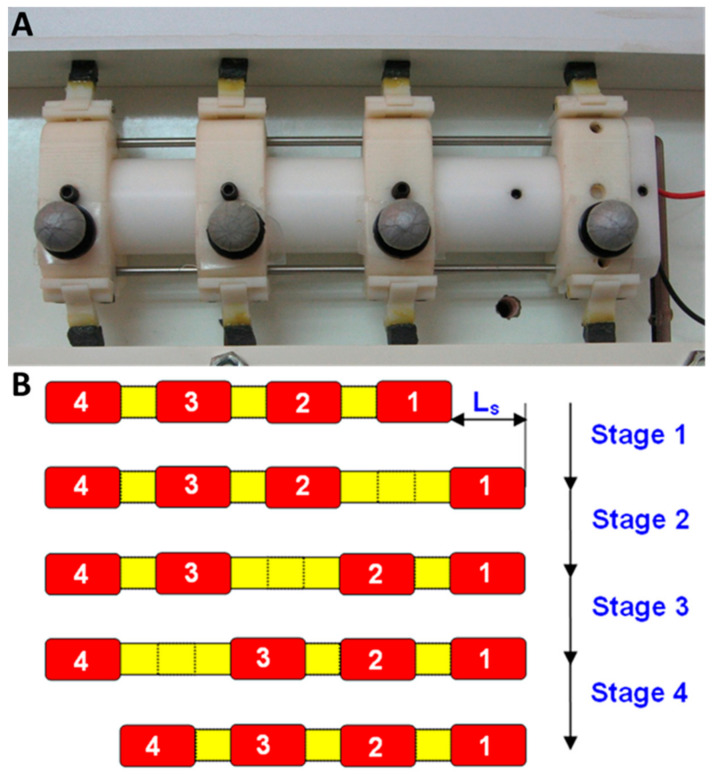
The worm robot used for the comparison (**A**) and its progress during one cycle of locomotion (**B**).

In this section, we make a comparison between the wave locomotion and worm locomotion. We use the numerical simulation to evaluate the speed of the wave robot and the analytical model developed in [24] to calculate the advancing distance of the worm in a single cycle of the worm. The worm robot was assumed to have four cells and a stroke of 7 cm (equivalent to the SAW’s advance in a single cycle on a rigid surface) and the COF value is one (similar to the actual experiment). Figure 19 presents the worm robot used for the comparison and its locomotion during one cycle of movement. During its movement, the robot systematically expands and contracts its gripping points on the surface.

**Figure 20:**
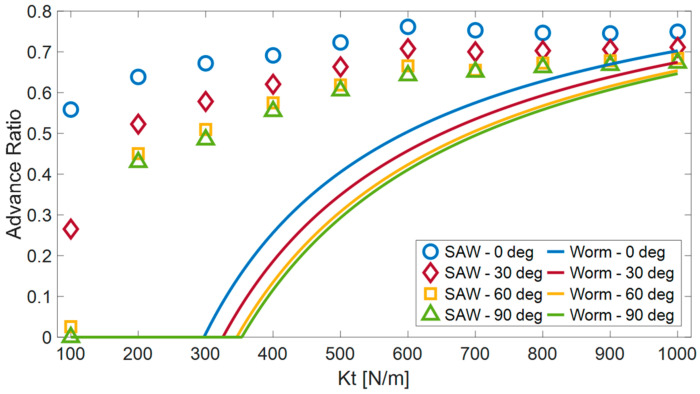
A comparison between worm and wave locomotion as a function of the surface compliance for four slopes.

In the first comparison (Figure 20), we analyzed the influence of surface compliance on the advance ratio for different climbing angles (0, 30, 60 and 90 degrees). In this experiment, the wave robot outperformed the worm robot in all the compliance values and the climbing angle. But the advantage reduced as the surface became more rigid.

The inability of the worm robot to move forward on surfaces with low tangential stiffness can be attributed to its method of locomotion. During each step of its movement cycle, the worm robot propels one segment forward, and the other segments are meant to exert counteracting forces to anchor the robot in position. When the surface’s stiffness is low, a relatively substantial deformation occurs in the surface to generate the necessary force. At this juncture, the robot effectively moves backward in tandem with the surface. As the surface’s flexibility increases, the retrograde motion of the robot becomes notably more pronounced, eventually culminating in a situation where no forward progress is achieved. In contrast, the motion of the wave robot is based on the continuous wave-like motion performed by its links. At any given moment, several links are stretching the surface in opposition to the direction of motion, creating a force that propels the robot forward. Low surface stiffness also affects the wave robot’s movement capability, although to a lesser extent.

**Figure 21:**
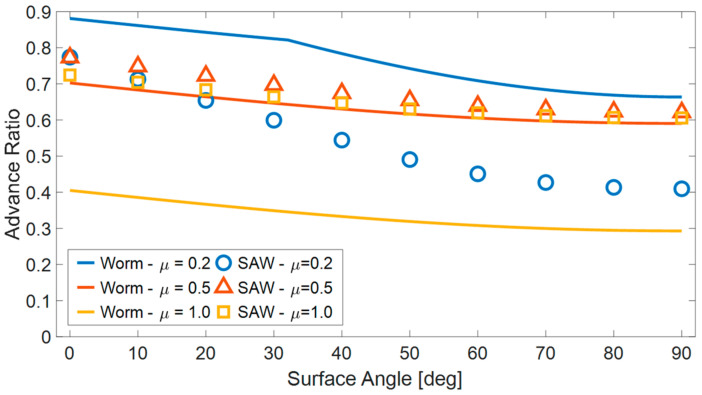
A comparison between worm and wave locomotion as a function of the climbing angle for three different COFs.

In the second comparison (Figure 21), we compared the AR of the worm locomotion to the wave locomotion as a function of the climbing angle for three different values of the COF. In this case, the wave achieved better performance for the higher COF values but its AR was lower for COF = 0.2.

The reason a higher COF hinders the worm robot’s ability to make progress is because, according to the model we used for comparison, as it advances its segments, there is still a certain degree of contact between them and the surface. As a result of this contact, a counteractive friction force is generated, opposing the segment’s movement, which pushes the robot backward. As the COF increases, this opposing force also increases, further impeding the robot’s forward motion.

In contrast, a higher COF aids the wave robot’s motion by enabling the application of larger tangential forces without slipping. However, it is important to note that increasing the COF could potentially hinder the robot’s progress. During the motion of the wave robot, multiple links touch the surface, and each link moves a distinct horizontal distance depending on its position within the wave cycle. When a link’s contact point with the surface has a smaller horizontal displacement than the robot’s actual advancement, it generates a resisting force that opposes the robot’s movement. Additionally, if other components of the robot, such as its head, touch the surface, they also exert a frictional force that acts against the robot’s forward movement. Therefore, there exists a trade-off between the assistance gained from higher COF values and the possible impediment they pose to the robot’s movement.

## 8. Conclusions

In this article, we examined the movement of a wave robot between two flexible surfaces against a resistant force and up a slope. The research was motivated by the potential use of these robots in medical applications, where they would need to move through biological vessels such as the intestines.

We started our analysis with a general description of the physics of locomotion for wave robots, which interact with the surface at multiple points. It became apparent that the interaction between the robot and the surface is continuous and indeterminate. Therefore, we needed to examine locomotion while considering the robot’s previous location, the rigidity of the surface, and its coefficient of friction.

To analyze the locomotion, we developed a numerical simulation that demonstrated that although the flexibility of the surface slows down the robot’s progress, the robot can still function effectively even in difficult situations, such as climbing vertically or when moving along very flexible surfaces. We compared these results to those of worm robots of a similar size and found that wave locomotion is superior to worm locomotion when the surface is highly flexible. For instance, when dealing with flexible surfaces exhibiting a stiffness below 300 N/m, the wave robot successfully advances and can even ascend slopes of up to 90 degrees. In contrast, the worm robot is unable to make any progress, even on a horizontal surface. An increase in pressure on the robot did not significantly decrease its ability to move forward, but this was not the case when the robot was climbing steep angles or moving vertically (unlike worm robots). The comparison results indicate that the worm’s motion outperforms the wave’s motion when the COF of the surfaces is low (COF = 0.2). Conversely, as the COF increases, the wave’s locomotion mechanism becomes more efficient, showing significant advantages for the wave when the COF reaches one. When crawling between surfaces with slopes ranging from 0 to 90 degrees, the wave robot achieved advance ratios (AR) ranging from 0.72 for horizontal movement to 0.61 for vertical climbing. In contrast, the worm robot demonstrated more modest AR values of 0.40 for horizontal movement and 0.29 for vertical climbing.

Finally, we created a miniature 3D-printed model of the robot to experimentally validate our analyses and simulations. The results of the experiments were in line with our expectations, as the robot crawled between the flexible surfaces as predicted by our analyses. The experimental results reveal that, as anticipated by the simulation, variations in the operating voltage of the motor and consequent changes in the wave’s motion speed have a minimal impact on the robot’s AR across all the examined surface angles. Furthermore, the simulation accurately predicted the experimental results, with an average relative error of approximately 11.37% between the AR values in the simulation and the experiments. The accuracy improves when considering higher operating voltages of 6 and 7 V, resulting in an average relative error of about 8.68%.

As is evident from the motion experiments (Figure 16) and the accompanying video attached to the manuscript, the robot’s movement displays a vibrational behavior oriented vertically in the direction of motion. These vibrations have an impact on the robot’s energy efficiency, and thus, reducing them would lead to an enhancement in efficiency. Our objective for future work is to mitigate these vibrations. This reduction can be achieved through various methods, including controlled motor activation with varying speeds and the utilization of a monolithic compliant robot structure [46].

## Data Availability

No new data were created or analyzed in this study. Data sharing is not applicable to this article.

## Supplementary Materials

The following supporting information can be downloaded at: https://www.mdpi.com/article/10.3390/biomimetics8050416/s1.

## Appendix A

### Appendix A.1. Robot’s Links Position and Orientation

The position of the front point (denoted with *F*) of each link (*i*) is identical to the rear point of the successive link:

(A1) $$\left(X_{i,F},Y_{i,F}\right)=\left(X_{i-1,R},Y_{i-1,R}\right).$$

The other four points defining the link’s position and orientation are calculated using the location of the front point, the geometrical properties of the links, and the propagated wave function. The center of the link is denoted with no additional index. The rear point is denoted with *R*, the upper point with *U,* and the bottom point with *D*. The coordinates of the center point are as follows:

(A2) $$\left(X_{i},Y_{i}\right)=\left(X_{i,F}-\sqrt{{\left(\frac{b}{2}\right)}^{2}-{\left(Y_{i}-Y_{i,F}\right)}^{2}},Y_{i,F}+\frac{b}{2}\frac{\psi \left(X_{i,F}\right)}{\sqrt{\psi^{2}\left(X_{i,F}\right)+1}}\right),$$

where *ψ*(*X_i_*) is defined as:

(A3) $$\psi \left(X_{i}\right)=Ak\sin\left[k\left(X_{1,F}-X_{i}\right)-\omega t\right].$$

The rear point’s coordinates are:

(A4) $$\left(X_{i,R},Y_{i,R}\right)=\left(X_{i}-\sqrt{{\left(\frac{b}{2}\right)}^{2}-{\left(Y_{i,R}-Y_{i}\right)}^{2}},Y_{i}+\frac{b}{2}\frac{\psi \left(X_{i}\right)}{\sqrt{\psi^{2}\left(X_{i}\right)+1}}\right),$$

the upper point’s coordinates are:

(A5) $$\left(X_{i,U},Y_{i,U}\right)=\left(X_{i}-\sqrt{r^{2}-{\left(Y_{i}-Y_{i,U}\right)}^{2}},Y_{i}+r\frac{\frac{1}{-\psi \left(X_{i}\right)}}{\sqrt{{\left(\frac{1}{-\psi \left(X_{i}\right)}\right)}^{2}+1}}\right),$$

and the bottom’s coordinates are:

(A6) $$\left(X_{i,D},Y_{i,D}\right)=\left(X_{i}+\sqrt{r^{2}-{\left(Y_{i}-Y_{i,D}\right)}^{2}},Y_{i}-r\frac{\frac{1}{-\psi \left(X_{i}\right)}}{\sqrt{{\left(\frac{1}{-\psi \left(X_{i}\right)}\right)}^{2}+1}}\right).$$

### Appendix A.2. The Pseudo Code of the Simulation’s Algorithm

The algorithm of the simulation is based on the following pseudo code:

1. Initialize surfaces’ form and robot position and orientation.
2. Calculate the links’ locomotion according to the wave function and the angular speed of the motor.
3. Verify no interference between the links during their locomotion.
4. For every time interval *dt* in the time range [*t_start_*, *t_end_*]:
  4.1. For every top or bottom tip point of the robot’s links:
    4.1.1. If there is contact with the surface, then:
      4.1.1.1. Calculate the degree of penetration into the surface (*δ_n_* = |Y*_surface_* − Y*_contact_point_*|).
      4.1.1.2. If there was contact in the previous frame as well, then:
        4.1.1.2.1. Calculate the change in the contact point location (Δ*_n_*, Δ*_t_*).
        4.1.1.2.2. If *δ_n,i_* > *δ_n,i_*_−1_
          4.1.1.2.2.1. *F_n_* = *k_n_*δ_n_*^1.5^
        4.1.1.2.3. Else,
          4.1.1.2.3.1. *F_n_* = *e*k_n_*δ_n_* ^1.5^
        4.1.1.2.4. *F*_*t*,*max*_ = *µ*F_n_*
        4.1.1.2.5. *Ft* = *k_t_**Δ*_t_*
        4.1.1.2.6. If *F_t_* < *F_t,max_*
          4.1.1.2.6.1. *δ_t_* = Δ*_t_*
        4.1.1.2.7. Else,
          4.1.1.2.7.1. *δ_t_* = *F*_*t*,*max*_/*k_t_* = *µ*F_n_*/*k_t_*
        4.1.1.2.8. *F_t_* = *k_t_*δ_t_*
      4.1.1.3. Else,
        4.1.1.3.1. *F_n_* = *k_n_*δ_n_* ^1.5^
        4.1.1.3.2. *F_t_* = 0
  4.2. Use the sum of forces and sum of moments equations to solve the linear and angular accelerations of the robot’s COM.
  4.3. Use numerical integration to find the linear and angular velocities, and the change in the robot’s angle and position.
  4.4. Update the robot’s position and orientation.
  4.5. Update the form of the surfaces according to the robot’s updated position.
